# Injuries to Nerves

**Published:** 1881-01

**Authors:** 


### INJURIES TO NERVES.

The following, from the proceedings of the St. Louis Medical Society, reported in the St. Louis Medical and Surgical Journal, on the reflex influence of injured and diseased nerves in connection with the teeth, is of especial interest to the dentist. Very considerable attention has been bestowed upon this subject, but much remains to be learned.—Ed.

Dr. Williams — I wish to refer to a case or two of reflex irritation that came recently under my observation. A few weeks ago a lady called at my office, saying that she had an earache for the past five or six days from which she could get no relief. I supposed before I looked into her ear that I would find either an abscess formed in the drum, or else an inflammation in the meatus. I found no evidence of inflammatory trouble in the ear at all. This led me to suppose that the source of the pain was not in the ear, but was reflected to it. I asked whether she had recently had toothache. She said that some time previously she had been troubled in that way, but that just then she was free from it. But at the time I saw her, her jaw was considerably swollen. Not finding any evidence of inflammatory trouble in the ear, and from the fact that her jaw was swollen, and that her teeth *had* been aching, I concluded at once that the cause of her trouble was in her teeth. I advised her to call on a dentist and have her teeth extracted. She did so, and the ear-ache immediately ceased.

A few days after that a colored woman came into my office with a little girl about twelve years old who had been suffering with an ear-ache for about two weeks. I expected to find inflammatory trouble in the ear, but to my surprise could discover none whatever. On making inquiry I found that the child had had toothache. Her jaw was swollen somewhat and tender, and I sent the child across the street to a dentist to have a tooth pulled. She called the next morning and said the pain had ceased immediately after the operation. These, I think, are striking cases of reflex irritation.

Some three or four years ago a lady of this city was suffering with neuralgia of her head and both sides of her face, Her suffering was intense. The weather was very cold and she supposed she had neuralgia of the whole scalp, and treated herself with the medicine that physicians prescribed, but without relief. Her suffering was so great that she could not bear contact with the cold atmosphere, and in order to protect herself from it she was accustomed to keep her head between two feather pillows. Even with that protection she suffered continuously. At this time I was treating her daughter for some affection of her eyes, and one day while in my office she described her mother's illness to me. I asked her whether her mother had not suffered also with her teeth. She replied that at that time she did not, but had previously been considerably troubled by them, and had suffered quite severely. I suggested that her mother should have all her tender teeth removed. This advice was followed, and immediately the neuralgia ceased and never returned.

Dr. Stevens — Dr. Williams in his remarks on reflex irritation refers to me a question in regard to the distribution of the sensitive nerves of the tympanic cavity. I do not think any one can understand reflex action without taking into consideration the anatomical connection of the nerves implicated.

The sensitive nerves of the tympanum, and structures of the internal ear, must be supplied from the tri-facial, in some way. I think the vidian nerve, as it passes backwards from the spheno-palatine ganglion, receives communicating branches from the tri-facial, and thus by its petrosal branch gives common sensation to the cavity. I conceive, also, that the corda tympani, from the seventh pair, may also carry some branches of the sensitive nerve. I do not know that it is any more mysterious that reflex irritation should affect the tympanum, or parts in immediate relation with it, than it is that irritation from a single tooth should be reflected to all the teeth, and in fact to the whole face.

Dr. Dickinson—A lady afflicted with partial blindness for several days, consulted me, I learned that she had a tooth filled recently, and that it was still tender. I told her that the tooth was the cause of disturbed vision, and that she would have to have it extracted. This she did, and on the following day came to my office with her vision perfectly restored.

